# Evaluation of correlated studies using liquid cell and cryo‐transmission electron microscopy: Hydration of calcium sulphate and the phase transformation pathways of bassanite to gypsum

**DOI:** 10.1111/jmi.13102

**Published:** 2022-04-18

**Authors:** M. Ilett, H. M. Freeman, Z. Aslam, J. M. Galloway, D. P. Klebl, S. P. Muench, I. J. McPherson, O. Cespedes, Y‐Y. Kim, F. C. Meldrum, S. R. Yeandel, C. L. Freeman, J. H. Harding, R. M. D. Brydson

**Affiliations:** ^1^ School of Chemical and Process Engineering University of Leeds Leeds UK; ^2^ The Bragg Centre for Materials Research University of Leeds Leeds UK; ^3^ School of Chemistry University of Leeds Leeds UK; ^4^ School of Biomedical Sciences and Astbury Centre for Structural and Molecular Biology University of Leeds Leeds UK; ^5^ Department of Chemistry University of Warwick, Gibbet Hill Coventry UK; ^6^ Department of Physics University of Leeds Leeds UK; ^7^ Department of Materials Science and Engineering University of Sheffield Sheffield UK

**Keywords:** calcium sulphate, cryogenic TEM, crystallisation, liquid phase TEM

## Abstract

Insight into the nucleation, growth and phase transformations of calcium sulphate could improve the performance of construction materials, reduce scaling in industrial processes and aid understanding of its formation in the natural environment. Recent studies have suggested that the calcium sulphate pseudo polymorph, gypsum (CaSO_4_·2H_2_O) can form in aqueous solution via a bassanite (CaSO_4_·0.5H_2_O) intermediate. Some in situ experimental work has also suggested that the transformation of bassanite to gypsum can occur through an oriented assembly mechanism. In this work, we have exploited liquid cell transmission electron microscopy (LCTEM) to study the transformation of bassanite to gypsum in an undersaturated aqueous solution of calcium sulphate. This was benchmarked against cryogenic TEM (cryo‐TEM) studies to validate internally the data obtained from the two microscopy techniques. When coupled with Raman spectroscopy, the real‐time data generated by LCTEM, and structural data obtained from cryo‐TEM show that bassanite can transform to gypsum via more than one pathway, the predominant one being dissolution/reprecipitation. Comparisons between LCTEM and cryo‐TEM also show that the transformation is slower within the confined region of the liquid cell as compared to a bulk solution. This work highlights the important role of a correlated microscopy approach for the study of dynamic processes such as crystallisation from solution if we are to extract true mechanistic understanding.

## 1 INTRODUCTION

Thanks to advances in analytical techniques, it is now recognised that many crystalline materials form via non‐classical nucleation and growth mechanisms.^1^ Liquid cell transmission electron microscopy (LCTEM) is becoming a key tool for characterising crystallisation mechanisms, where it enables crystallisation processes to be visualised in solution in real time and at high resolution.^2^, ^3^, ^4^ This gives unique insight into crystallisation pathways and the early stages of crystallisation processes. However, LCTEM is a complex technique. Electron beam effects can change the chemistry of the liquid; for example, radiolysis of water yields hydrated electrons (*e_h_*) and OH˙, H˙ and H_2_˙ radicals. These species react to produce H_2_O_2_, H_3_O^+^ and H_2_O^–^, which can further react with other species in the solution.^5^, ^6^, ^7^, ^8^ Such chemical changes mean that observations are not true representations of ‘bulk’ reactions. The liquid within the LC is also highly confined, typically <2 nL, which can further influence observations. It is widely acknowledged that confinement can have a significant impact on fluid mixing, crystal nucleation rates, polymorph formation, morphologies and orientations.^9^

Cryogenic (cryo‐)TEM has also been widely used to study crystallisation processes and offers some advantages over LCTEM. Rapid freezing of the sample entraps it within vitreous ice in a state analogous to that in solution. In this frozen state, it is possible to perform detailed analysis at specified reaction times. Electron beam effects are also reduced, and so too are confinement effects. LCTEM and cryo‐TEM are therefore complementary, and together offer a powerful means of studying crystallisation processes.

This work combines LCTEM and cryo‐TEM to study the mechanisms by which bassanite (CaSO_4_·0.5H_2_O) transforms to gypsum (CaSO_4_·2H_2_O) in aqueous solution. Calcium sulphate is important in both environmental and engineering contexts and exists as anhydrite (CaSO_4_), bassanite and gypsum. Gypsum is the most stable phase under ambient conditions, while anhydrite becomes stable at temperatures above *ca*. 60°C. Bassanite is always unstable with respect to anhydrite and gypsum but precipitates from solution at temperatures over *ca*. 90°C due to the slow kinetics of anhydrite formation.^10^ Work performed over the last 10 years, which shows that bassanite can precipitate from aqueous solutions at room temperature, therefore contradicts this picture.

Significant recent efforts have been made to determine the mechanisms by which calcium sulphate precipitates from aqueous solution. Wang *et al*.^11^ reported an amorphous precursor phase during the precipitation of gypsum from aqueous solution, an observation that was subsequently confirmed by others.^12^, ^13^, ^14^, ^15^ They also observed that bassanite formed prior to gypsum in solutions of concentration 15–50 mM, although ethanol (which can promote the formation of bassanite over gypsum) was used to wash the samples. Van Driessche *et al*.^16^ also identified bassanite as an intermediate in gypsum formation, using vacuum/solvent filtration and cryogenic quenching to prepare samples from solution concentrations of 50–150 mM. They suggested that gypsum formed via self‐assembly of bassanite nanocrystals co‐oriented along the *c* axis of bassanite. However, subsequent in situ small‐angle X‐ray scattering studies by Stawski *et al*.^17^ failed to identify a bassanite precursor to gypsum at the same concentrations. When couple with molecular dynamics (MD) simulations, their data suggested a four‐stage model whereby sub‐3 nm nuclei form, assemble into domains which then densify, before transforming to gypsum through orientated attachment. Recent TEM studies of gypsum formation from 50 mM solution concentrations at room temperature in which samples were isolated by vacuum filtration also suggested a multi‐stage process where bassanite nanorods and rosettes grew by aggregation or ion addition, and gypsum then nucleated on the tips of the bassanite needles.^18^

The diversity of mechanisms observed is at least in part due to the differing methods of sample preparation and characterisation techniques employed, which could be unintentionally altering the specimens. Mineral phases often change on drying or by the addition of solvents, and artefactual assembly of particles can occur as well as changes to the actual kinetics of any observed process. in situ studies eliminate many of these problems, but changes can still be induced during characterisation. Here, we combine LCTEM, cryo‐TEM and in situ Raman spectroscopy to study the transformation of bassanite to gypsum in an undersaturated aqueous solution. The decision to monitor the transformation of one solid phase to another in the presence of a controlled liquid environment, rather than the full crystallisation of gypsum from ions in solution, simplifies some of the complexities of LCTEM experiments (e.g. preferential heterogeneous nucleation on the windows of the liquid cell). This allows us to benchmark our LCTEM observations against other techniques and sample preparation methods and reveals that bassanite primarily transforms to gypsum via a dissolution/reprecipitation mechanism in our particular system.

## 2 RESULTS

### 2.1 in situ observation of the transformation of bassanite to gypsum using Liquid cell TEM

**FIGURE 1:**
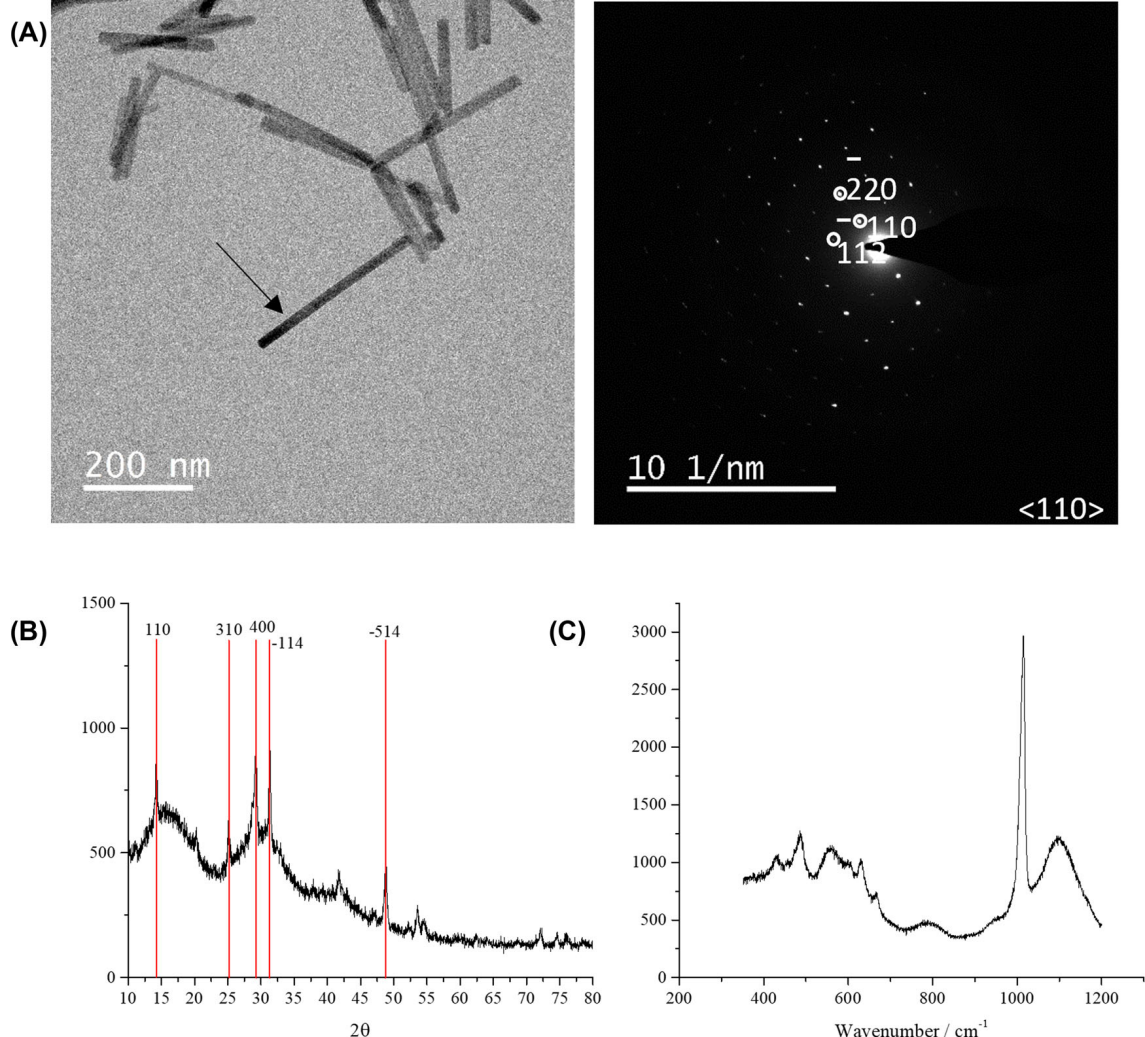
Characterisation of synthesised bassanite nanorods. (A) TEM image showing rods of 200–400 nm in length and SAED of the particle indicated by the arrow was indexed to bassanite. (B) XRD analysis shows characteristic peaks at *d*_110_ 6.00 Å, *d*_310_ 3.47 Å, *d*_400_ 3.00 Å, *d*_–114_ 2.80 Å, *d*_–514_ 1.85 Å. (C) Raman spectroscopy indicates strong peaks at 1015 cm^−1^ associated with the *υ*_1_ SO_4_ band along with peaks at 427, 489, 628, 668 and 1128 cm^−1^ associated with the *υ*_2_/ *υ*_3_/ *υ*_4_ (SO_4_) vibrational modes (see Table 1)

Bassanite nanorods were synthesised by combining equal volumes of 50 mM aqueous solutions of CaCl_2_ and (NH_4_)_2_SO_4_, and then rapidly quenching the mixture in an excess of ethanol as per Tritschler *et al*.^19^ The product nanorods were ca. 300 ± 150 nm in length and selected area electron diffraction (SAED), X‐ray diffraction (XRD) and Raman spectroscopy confirmed that they were bassanite (Figure 1, Table 1). Real‐time videos of the transformation of these nanorods to gypsum were recorded using LCTEM, where scanning TEM (STEM) was used rather than conventional (parallel beam) TEM (CTEM) as it provides better contrast and therefore resolution when imaging thicker liquid regions. Electron flux/fluences also tend to be higher in CTEM, which can lead to more pronounced bowing of the windows in the LC cell or alternatively snapping shut of the windows which then expels the liquid from between the two LC chips. Transformation of bassanite to gypsum was induced by flowing through an undersaturated aqueous solution of CaSO_4_ (9:1 [12 mM CaSO_4_(aq)]:[ethanol]). See Section 5 for more details.

**TABLE 1:** Wavenumbers (cm^−1^) of Raman sulphate ion modes for bassanite, gypsum and (SO_4_^2–^)_aq_

Band	Bassanite	Gypsum	(SO_4_^2–^)_aq_
*υ*_2_ δ_symm_ (SO_4_^2–^)	430	415	451
	489	493	
*υ*_4_ δ_asymm_ (SO_4_^2–^)	629	619	613
	668	670	
*υ*_1_ *υ*_symm_ (SO_4_^2–^)	1016	1008	981
*υ*_3_ *υ*_asymm_ (SO_4_^2–^)	1116	1117	1104
	1129	1135	
	1151		
	1167		
	1181		

**FIGURE 2:**
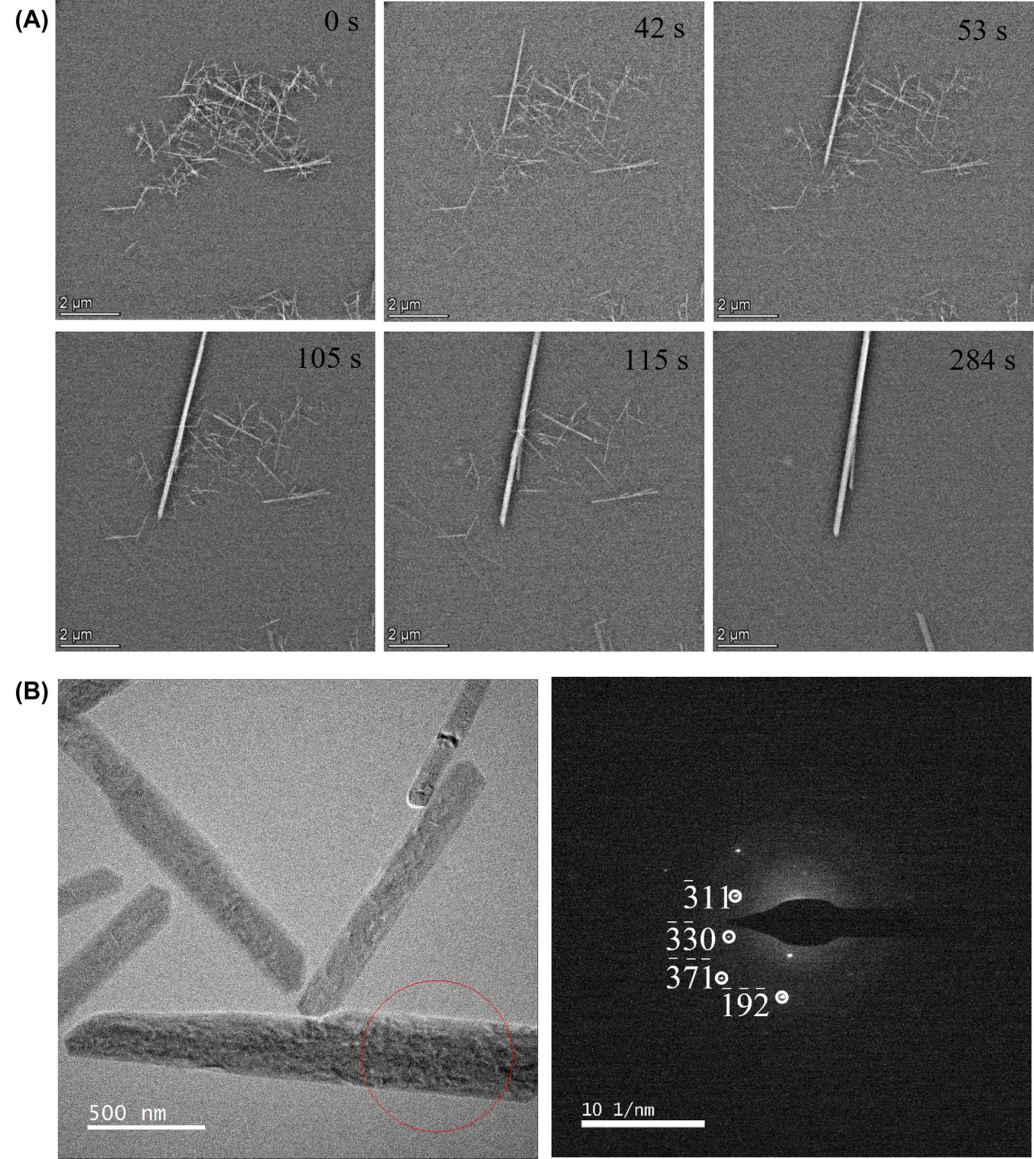
(A) Image series taken from Video S1 at different time points showing the transformation of bassanite to gypsum through hydration using a 9:1 [12 mM CaSO_4_(aq)]:[ethanol] solution. Dissolution of bassanite occurred followed by gypsum nucleation and continued dissolution and reprecipitation of bassanite on growing gypsum crystals. All bassanite nanorods transformed after ∼200 s. (B) Post‐mortem SAED analysis carried out after pushing air through the LC confirmed gypsum crystals had been formed. The circled region indicates the region from which the SAED pattern was obtained and the scale bar is 500 nm

These experiments showed that the bassanite to gypsum transformation occurred predominantly via dissolution of bassanite nanorods before nucleation and growth of less soluble gypsum crystals (Figure 2 and Video S1). Real‐time observation of this process indicated that the bassanite nanorods preferentially dissolve from the ends of the rods. Once gypsum begins to nucleate then all the remaining bassanite dissolves and reprecipitates as gypsum on the growing gypsum crystals. We assume that heterogeneous nucleation of gypsum initially occurs on either the dissolving bassanite particles (although not observed at this magnification) and/or the LC chip windows. Typically, the bassanite transformation occurred on ∼100 s timescales, where time zero is the point at which the undersaturated aqueous CaSO_4_ solution is flowed through the LC to initiate the reaction. Figure 2A shows snap shots from Video S1 where initial gypsum nucleation is observed at 42 s. Gypsum growth then occurs simultaneously with continued bassanite dissolution until at ∼ 284 s only gypsum crystals are observed (identified from the distinct difference in their morphology when compared to the initial bassanite nanorods). Beyond this time point, no changes to the observed crystals are seen. Post‐mortem SAED analysis (i.e. after removing the liquid from the LC) of the end‐product confirmed that it was indeed gypsum that had formed (Figure 2B) with distinctive ∼3 Å *d*‐spacings observed matching the (041) lattice plane. This experiment was repeated numerous times and on occasion limited rearrangement of the bassanite rods prior to transformation into gypsum was observed: For example, in Video S2, some gypsum appears to form without an initial dissolution step (see the region towards the bottom left). However, this could have been induced by dissolution of bassanite from beyond the field of view. Overall, the LCTEM analysis conclusively demonstrates that a dissolution/reprecipitation mechanism dominates in this environment.

**FIGURE 3:**
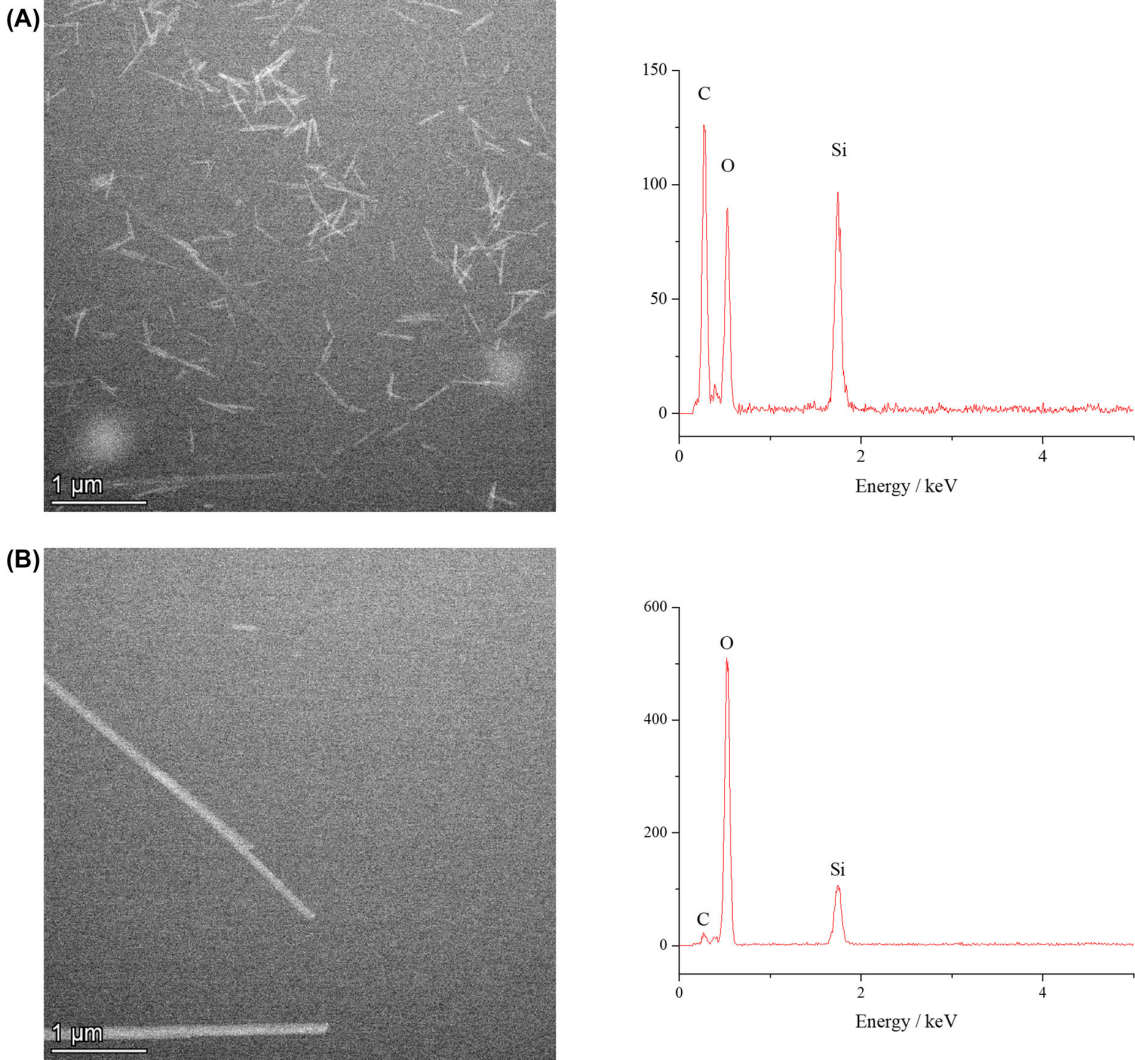
Verification that the transformation of bassanite to gypsum was not solely beam induced. (A) Initial HAADF STEM image of bassanite nanorod precursor (5.8 *e* ^–^/Å^2^) alongside EDX spectra showing large C Kα peak indicative of the nanoparticles being dispersed in ethanol. (B) HAADF STEM image taken after 5 min of flowing a 9:1 [12 mM CaSO_4_(aq)]:[ethanol] solution though the LC chip and an additional 2 min with beam blanked, alongside EDX spectra clearly showing a large O Kα peak and reduced C Kα peak indicative of the cell being filled with an aqueous solution

Additional studies were undertaken to interpret any electron beam contributions to the transformation process. For this, an initial image of bassanite in ethanol in the LC was taken with as little exposure to the electron beam as possible (Figure 3A). The beam was then blanked and the 9:1 [12 mM CaSO_4_(aq)]:[ethanol] solution was flowed into the LC. After flowing through the aqueous CaSO_4_ solution for 1 min to ensure the LC was now an aqueous environment the flow was stopped, and the system left for 2 min still with the beam blanked. The beam was then unblanked and a final image recorded (Figure 3B). Gypsum crystals had formed in the absence of the *e* ^–^ beam, showing that the overall transformation from bassanite to gypsum was not beam‐induced. Complementary energy dispersive X‐ray (EDX) analysis before and after the reaction also confirmed the initial ethanol content (high carbon detected) and the final aqueous (low carbon detected) content of the LC. Furthermore, extended exposure of the initial bassanite nanorods in ethanol in LCTEM showed they were stable to the electron beam (Figure S1). We note that the presence of ethanol can act as a scavenger, which may reduce the effects of the electron beam radiolysis of water during our LCTEM analysis.^20^

**FIGURE 4:**
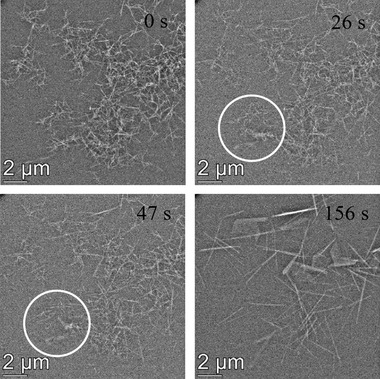
Images taken from Video S3 where ethanol was preflowed through the LC to ‘loosen’ the bassanite nanorods prior to the reaction taking place. Some movement of the nanorod precursor was observed within the circled regions, but the overall mechanisms of transformation was via dissolution and reprecipitation

Finally, the possibility that the liquid cell constrains any movement of the bassanite nanorods was also considered as this may affect the observed transformation pathway. The LC chip window once loaded with bassanite nanorods was plasma‐treated prior to assembly of the cell to give sufficient loading of the bassanite nanorods, and this could potentially constrain their motion by drying them onto the LC window. Continued scanning over an area of bassanite crystals prior to flowing through the CaSO_4_(aq) solution showed no obvious movement of the crystals, suggesting they were firmly adhered to the window rather than free to move about in the ethanol solution (Video S3). Further ethanol was therefore initially flowed through the chip to ‘loosen’ the bassanite rods from the window prior to carrying out the hydration experiment. This appeared to provide some freedom of movement to the bassanite nanorods evidenced in Video S4 (towards the bottom left) and the circled regions in Figure 4, but the majority of nanorods remained stuck to the window. Notably, these particles were still observed to transform to gypsum via a dissolution/reprecipitation mechanism.

### 2.2 in situ observation of the transformation of bassanite to gypsum using Raman spectroscopy

**FIGURE 5:**
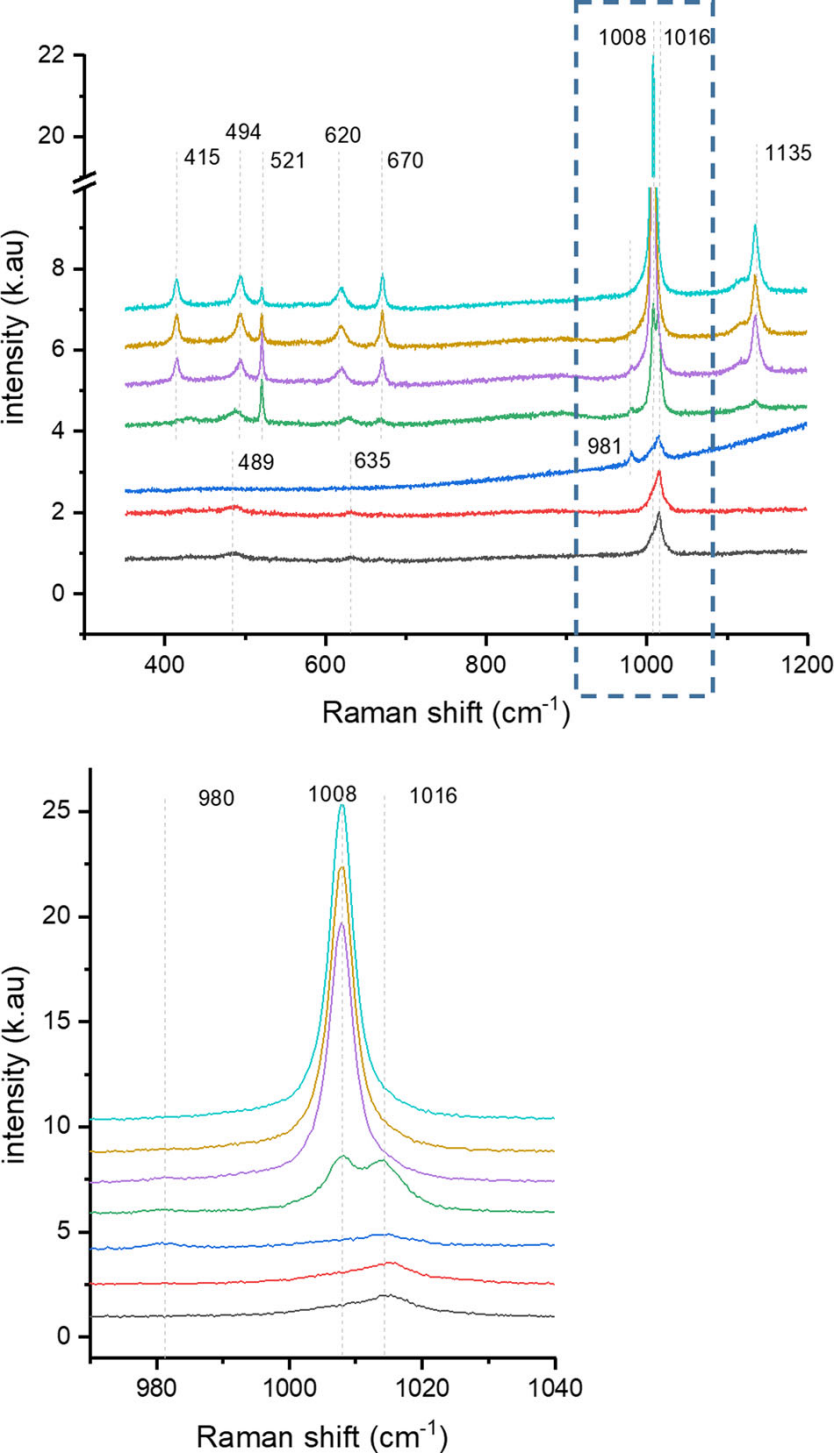
in situ Raman spectral series of the transformation of bassanite nanorods exposed to an undersaturated CaSO_4(aq)_ solution inducing hydration and a transformation to gypsum. The bottom spectrum is the area highlighted in the dashed box in the upper spectrum. The coloured lines denote the varying time points where, black refers to time = 0 s; red, time = 90 s; blue, time = 180 s; green, time = 270 s; purple, time = 360 s; gold, time = 450 s and cyan, time = 540 s

Raman spectroscopy was also used to monitor the transformation of bassanite to gypsum, where the in situ experiment was conducted by placing the LC holder tip within a Raman microscope. The time‐resolved Raman spectral series recorded is presented in Figure 5 and the wavenumbers of the Raman modes associated with bassanite and gypsum are shown in Table 1. The initial bassanite *υ*_1_ *υ*_symm_ (SO_4_^2–^) band at 1016 cm^−1^ remains constant until 90 s. After 180 s, this peak decreased in intensity and an additional peak at *ca*. 980 cm^−1^ appeared due to aqueous sulphate (*υ*_1_ *υ*_symm_ (SO_4_^2–^) band), consistent with dissolution of the bassanite. Peaks specific to gypsum then appeared by 270 s, including the gypsum *υ*_1_ *υ*_symm_ (SO_4_^2–^) band at 1008 cm^−1^ and no bassanite was visible by 360 s.

### 2.3 Observation of the transformation of bassanite to gypsum using cryo‐TEM

Initial cryo‐EM experiments were carried out using conventional plunge freezing protocols to capture snapshots of the hydration of bassanite at specific time points. Transformation to gypsum was observed after ∼20 s (Figure S2). In order to probe shorter time points so as to correlate with LC observations, a home‐built spray plunge freezing machine was used which allows spray sample application and plunge freezing at much shorter time points than are achievable using a manual vitrobot set‐up.

Early reaction times were studied as follows: (i) the bassanite nanorods were dried on a TEM grid and then the 9:1 [12mM CaSO_4(aq)_]:[EtOH] solution was sprayed onto it (on‐grid mixing) or (ii) the bassanite and 9:1 [12mM CaSO_4(aq)_]:[EtOH] solutions were premixed and sprayed simultaneously onto the grid, ensuring free movement of the nanorods during rehydration (in‐flow mixing). See Section 5 for more details.

**FIGURE 6:**
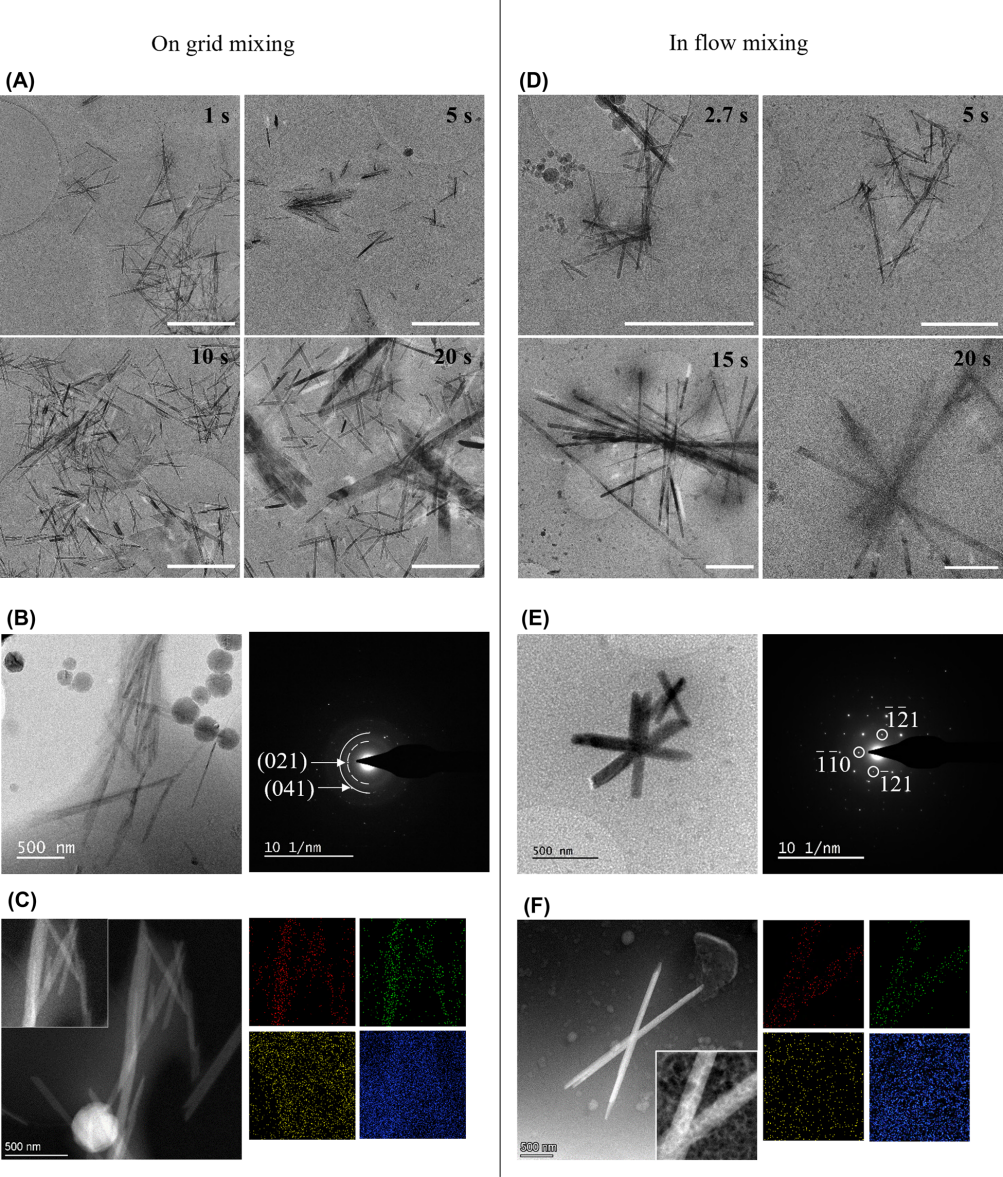
(A) Time resolved cryo‐EM images taken at 1, 5, 10 and 20 s after spraying aqueous 9:1 [12 mM CaSO_4_(aq)]:[ethanol] on a TEM grid loaded with predried bassanite nanorod precursor (on‐grid mixing) (scale bar 1 μm). Transformation to gypsum appears to occur between 5 and 10 s. Gypsum crystals were confirmed by SAED showing distinctive rings at 3 Å (solid line) and 4.2 Å (dashed line) *d*‐spacings attributed to the (041) and (021) lattice planes respectively (B). EDX confirms Ca (red) and S (green) rich crystals (C). Spherical particles observed in S/TEM images are frost contamination. (D) Time resolved cryo‐EM images taken at 2.7, 5, 15 and 20 s after mixing aqueous 9:1 [12 mM CaSO_4_(aq)]:[ethanol] and bassanite nanorods in ethanol and spraying onto a plasma cleaned TEM grid (in‐flow mixing) (scale bar 1 μm). Gypsum begins to form after 15 s. Gypsum crystals were confirmed by SAED showing distinctive 4.7 Å *d*‐spacing (‐111) (E). EDX confirmed Ca (red) and S (green) rich crystals, oxygen (blue) carbon (yellow) (F)

Figure 6 compares the two different processes. For on‐grid mixing, the reaction was stopped at 1, 5, 10 and 20 s after spraying the 9:1 [12mM CaSO_4(aq)_]:[EtOH] solution onto the preloaded TEM grid. Shorter bassanite nanorods were the predominant crystal observed at 1 and 5 s, while a significant morphological change was observed after 10–20 s to larger crystals indicative of gypsum. This was confirmed using SAED, which showed distinctive rings at 4.2 and 3 Å *d*‐spacings for the crystals matching to the (021) and (041) lattice planes respectively (Figure 6B), and also STEM/EDX (Figure 6C) which showed Ca‐ and S‐rich crystals. For in‐flow mixing, the sample was frozen after median time points of 2.7, 5, 15 and 20 s. Again, the majority of crystals after 2.7 and 5 s were observed to be bassanite, and a significant morphological change occurred after 15–20 s with SAED and STEM/EDX confirming the larger crystals were indeed gypsum (Figure 6E and F). However, qualitative assessment of the images suggested fewer of the precursor bassanite nanorods had transformed to gypsum after 15 and 20 s for the in‐flow mixing than for on‐grid mixing. This is expected due to the differences in the aqueous content, where in‐flow mixing results in a ∼45% H_2_O concentration as compared to ∼90% for the on‐grid mixing, so speeding up the kinetics of the hydration process for on‐grid mixing. In addition, notably fewer crystals were present on grids prepared by in‐flow mixing, which is again expected due to a smaller volume sprayed onto the grid and lower binding than compared to conventional droplet loading onto a TEM grid.

No evidence for the oriented attachment of bassanite needles and subsequent solid‐state transformation into gypsum was obtained on qualitative evaluation of these images. The images were also subjected to quantitative analysis using the directionality plugin in ImageJ^21^ which calculates the orientation of particles within an image relative to a horizontal line across the image. Peaks appear in the directionality plot if the particles are aligned, as compared with a flat line for randomly orientated particles. The directionality plots show little evidence for any alignment of the bassanite nanorods for either in‐flow or on‐grid mixing (Figure S3A and B) with both plots showing relatively flat lines within error. For in‐flow mixing some peaks were observed at 20 s; however, this would be expected where there are fewer larger gypsum crystals within the images. A direct comparison of the same 5 s time point illustrated no significant differences between the two mixing methods (Figure S3C).

Therefore, considering the analytical TEM data and directionality analysis, correlative cryo‐EM studies appear to confirm the observation of LCTEM that the predominant pathway of transformation is via a dissolution/reprecipitation process.

## 3 DISCUSSION

### 3.1 Implications for in situ microscopy

Sample preparation and loading for the LC experiments were carefully evaluated in order to minimise any effect on the observed transformation pathway. If the plasma cleaning step following loading of the ethanol bassanite suspension onto the LC chips was omitted, little to no bassanite was observed in the LC window. However, plasma cleaning in order to ‘stick’ the rods to the window raised a concern regarding the removal of any free movement of bassanite within the LC environment. As an alternative, flowing ethanol through the LC chip prior to the experiment only released the bassanite nanorods to a limited extent, with the majority remaining adhered to the LC window. The two different time‐resolved cryo‐TEM methods were carried out in part to ensure no artefacts from plasma cleaning were observed in the LCTEM experiments. On‐grid mixing was a like‐for‐like comparison with the LC experiments, whereby the bassanite was ‘stuck’ to the TEM grid before aqueous CaSO_4_ was sprayed onto it and the transformation allowed to take place with bassanite having no freedom of movement. Alternatively, in‐flow mixing allowed the free movement of bassanite during the hydration process. Importantly no significant differences were observed between the on‐grid and in‐flow mixing following cryo‐TEM image analysis, and significantly, the transformations observed in both the LC and cryo‐TEM experiments were in agreement, indicating that the plasma cleaning step required for the LCTEM experiment did not alter the mechanism of transformation observed or force any artefactual misinterpretation of results. Independent of whether the precursor bassanite nanorods were fixed or in suspension, the predominant pathway of hydration was by a dissolution and reprecipitation mechanism.

Further problems in interpreting LCTEM observations arise from complicated electron beam induced effects. Here, using simple beam blanking experiments, we have shown that the overall transformation of bassanite to gypsum was not inherently beam induced. However, there were considerable kinetic differences between the transformation that was observed using LCTEM and Raman spectroscopy within the same in situ cell. Observing the transformation using in situ Raman spectroscopy, the kinetics of the reaction were slower than observations from LCTEM experiments, wherein gypsum forms after ∼270 s for Raman spectroscopy and ∼100 s for LCTEM. Modelling has shown that dose rates (electron fluxes e/(Å^2^ s)) of the order of 10^3^ Gy/s can cause significant decreases in pH for aqueous solutions within the liquid cell environment.^8^ As 1 *e* ^–^/(Å^2^ s) is equivalent to 1.2–3.6 MGy/s when irradiating water in a LCTEM cell at 300 kV, considerable pH changes would be expected even at very low electron flux.^22^ For the LC experiments carried out in this research, electron fluxes between 0.14 and 0.032 *e* ^–^/(Å s) were used, which were required to ensure sufficient contrast when imaging through a 250 nm layer of liquid. This is of the order of 10^5^ Gy/s and would therefore be expected to reduce the pH of the system. For Raman spectroscopy there are few beam induced effects since the photon irradiation does not induce water radiolysis^23^ and consequently, there will be limited if any reduction in pH. Thus the difference in kinetics between Raman spectroscopy and LCTEM is attributed to the pH reduction in LCTEM which accelerates bassanite dissolution and subsequently the transformation kinetics.^24^ A related observation in LCTEM experiments was that as the bassanite nanorods dissolved rapidly at the low pH induced by the electron beam, the solubility limit was exceeded and, following precipitation of gypsum crystals, what we assume were amorphous calcium sulphate particles were observed to precipitate from solution (Figure S4). This phenomenon appeared to be induced by prolonged exposure of the electron beam and took longer at lower undersaturations, for example, 12 mM solutions as compared to 15 mM.

Additional kinetic differences were also noted between the correlative cryo‐TEM and LCTEM studies. There was a discernible difference in the rate of transformation of bassanite, where transformation to gypsum typically started after just 10 s for cryo‐TEM, as compared to LCTEM experiments where the corresponding time was ∼100 s (and even longer for the in situ Raman measurements in the same confined chip). Such differences are attributed to variations in the degree of confinement within the two techniques. The thin layer of vitreous ice within cryo‐TEM does itself result in a confined environment and this has been shown previously by Ten Hove *et al*.^25^ to cause artefactual size‐sorting across holes on the TEM grid, where large particles tend to sort to the outer (thicker) regions of the hole and smaller particles are found closer to the central (thinner) regions. Such size‐sorting could be important in particle aggregation mechanisms (where smaller particles preferentially aggregate over large ones). However, here we did not observe any significant evidence of this phenomenon across holes on the cryo‐TEM grid and, furthermore, the blotting process which is suggested to be an important factor in size‐sorting was not used in the cryo‐TEM time‐resolved spray setup. Comparatively, the environment within the LC is much more confined than in cryo‐EM and is present throughout the whole crystallisation process. Therefore, the slower reaction kinetics observed in LCTEM are expected and this is fully consistent with the literature which reports that crystal nucleation and growth are retarded in small volumes.^9^ ^,^ ^26^, ^27^, ^28^ Previous studies of calcium sulphate in confinement have shown that stabilisation of amorphous calcium sulphate and bassanite occurs even at the micrometre scale.^29^ Stabilisation of bassanite for over 3 weeks was also reported in nanoscale pores.^15^ Furthermore, there is ongoing discussion regarding the homogeneity of mixing during flow within the LC chip. In the set up here, bassanite is initially dispersed in 100% ethanol before the 9:1 [12 mM CaSO_4_]:[ethanol] solution is flowed through the LC. At the initial point of mixing (which occurs some distance from the observed electron transparent window), there will still be an excess of ethanol that will stabilise the bassanite nanorods for some period of time following the start of the flow of aqueous calcium sulphate solution through the cell. This may also increase the time for hydration to take place, as ethanol can stabilise bassanite with respect to gypsum.^19^ Mixing on the TEM grid for the cryo‐TEM experiments is expected to be more homogeneous, which would be expected to speed up the transformation of bassanite to gypsum.

Overall, our data highlights the importance of carrying out combined EM techniques in order to better understand reaction mechanisms. Wang *et al*.^29^ recently used a similar ‘distributed EM’ process to study the desilication of zeolite crystals. Their work also emphasised the importance of correlating LC and cryo‐EM work in order to rule out any electron beam effects that are more dominant in LCTEM and came to the same conclusions in terms of the kinetic differences between LC and cryo‐TEM, in that the confinement within the liquid cell reduces the reaction kinetics, whereas conversely the radiolytic effect of the electron beam on the liquid water can increase the reaction kinetics.

### 3.2 Implications for the hydration pathway of calcium sulphate

Our data clearly show that bassanite nanorods transform to gypsum via a dissolution/reprecipitation pathway within a controlled liquid environment, where this was confirmed by complementary in situ Raman and cryo‐TEM studies. This is consistent with the literature describing the hydration of plaster of Paris (bassanite) for applications within the construction industry.^30^, ^31^, ^32^, ^33^ No evidence was obtained for the oriented assembly of bassanite nanorods prior to direct transformation into gypsum. The latter mechanism has been reported in a number of studies,^14^ ^;^ ^19^ ^;^ ^16^ but it is notable that most of those analyses were performed *ex situ*. It is possible that the ethanol in the mixing solution in our LCTEM studies inhibited oriented attachment,^34^ yet notably, the nanorods employed here are significantly larger than the nanorod precursors described in the papers of Stawski *et al*.^17^, ^35^ Since oriented attachment of crystalline nanoparticles is driven by the reduction in surface energy,^36^ it is therefore typically only observed for particles <10 nm in size and consequently we would thus not expect it to dominate in our system.

Finally, insight into the dissolution/reprecipitation mechanism is obtained from forthcoming related work by Yeandel *et al*.^37^ where hydrated surface‐free energies in bassanite were calculated using recently developed MD methods. These calculations confirm the needle‐like morphology of bassanite observed in this work and also reported by Kong *et al*.^38^ for bassanite grown in reverse microemulsions with high concentrations of sodium dodecyl sulphonate. The needles exhibit {110} and {100} side facets and are elongated along the <001> direction. The MD calculations indicate strong binding of water to the {110} face which stabilises this surface, although strong water layering also reduces entropy which will counteract this effect. Nevertheless, bassanite {110} (and related surfaces) are still the most stable, with the {001} surfaces capping the rods. The {001} surface of bassanite also exposes channels for ingress of water into the structure. Consequently, any preferential dissolution of the bassanite should occur from the ends of the rods as was observed in this work (Video S1).

The {001} surface of bassanite also presents some evidence of epitaxy with the {001} surface of gypsum since both exhibit a hexagonal motif of calcium and sulphate ions, and chains of Ca‐SO_4_ perpendicular to {001} (Figure S5). The major difference between the gypsum and bassanite structures is the elongation of gypsum along its *b*‐axis (by ∼25%) due to the inclusion of planes of water. This potentially low‐energy bassanite–gypsum interface, which may in reality be non‐stoichiometric in terms of water content so improving any epitaxial matching of structures, would then potentially favour the heterogeneous nucleation of gypsum on the {001} tips of the dissolving bassanite needles, as suggested by Jia *et al*.^18^ However, such heterogeneous nucleation of gypsum was not directly observed here in our liquid cell TEM or cryo‐TEM experiments.

## 4 CONCLUSIONS

This work presents a correlative study of the transformation mechanism of bassanite to gypsum using in situ Raman spectroscopy, in situ LCTEM and time‐resolved cryo‐TEM. The dominant pathway for the transformation of ∼300 nm bassanite nanorods to gypsum is shown to be a dissolution/ reprecipitation process whereby we suggest gypsum crystals nucleate on dissolving bassanite nanorods and subsequently grow. This work highlights the complexity of this microscopy‐based study, where some discrepancies in the mechanistic pathways and kinetics were apparent between the different techniques. Notably, the confinement within the microfluidic environment of the LC chip used for the LCTEM and Raman studies slowed the reaction as compared with bulk solution. Conversely, the radiolysis of the aqueous solvent and concomitant reduction in pH that occurs during LCTEM observation accelerated the reaction due to increased bassanite dissolution. These issues highlight the importance of carrying out correlative studies using multiple in situ techniques, where we recommend that LCTEM and cryo‐TEM are used in combination. Ultimately, these cutting‐edge microscopy techniques hold great promise in the complex field of crystallisation and phase transformations more generally, and we next intend to apply the knowledge gained in this work to the study of the direct formation of gypsum from aqueous solution using the correlated methods outlined above.

## 5 MATERIALS AND METHODS

### 5.1 Synthesis of the precursor material and undersaturated hydrating solution

The bassanite nanorod precursor was synthesised following a procedure adapted from Tritschler *et al*.^19^ In short, equal volumes (2.5 ml) of aqueous 50 mM CaCl_2_ and 50 mM (NH_4_)_2_SO_4_ were prepared and then mixed briefly before adding to 45 ml of ethanol. This was left for 5 min to allow bassanite to form. The mixture was then centrifuged at 6000 rpm for 1 min, the supernatant removed and the bassanite redispersed in 5 ml ethanol to concentrate the solids and stabilise the bassanite. Size analysis of the bassanite nanorods was carried out on >100 particles using Gatan Microscopy Suite software, DigitalMicrograph®.

Preliminary studies were carried out to ascertain the optimal aqueous CaSO_4_ solution to flow through the LC chip to initiate the bassanite to gypsum transformation. Initially the concentration of the CaSO_4_ aqueous solution was investigated, varying it between 10 and 15 mM, and it was decided to use a slightly undersaturated 12 mM solution for optimised experiments. 100% water resulted in a hydration and dissolution process that was too quick to monitor adequately. Therefore, a 12 mM aqueous CaSO_4_ solution was used with an additional 10% ethanol content. With higher ethanol content (80% and 60%), no reaction was observed suggesting the water content was too low. Therefore to induce the transformation of bassanite to gypsum, a solution of 9:1 [12 mM CaSO_4_(aq)]:[ethanol] was used.

### 5.2 Liquid Cell TEM

A dual flow Hummingbird Scientific liquid cell TEM holder was used for in situ experiments. A schematic of the holder is presented in Figure S6 where a thin liquid layer is encased by two silicon nitride chips with 250 nm spacers and 200 μm × 50 μm × 50 nm silicon nitride windows. Before loading the liquid cell holder, the chips were plasma cleaned for 60 s using an Ar/O_2_ gas mixture (Henniker plasma HPT‐100), and 2 μl of bassanite nanoparticles (sonicated for 5 min and dispersed in ethanol) were dispensed onto the top chip. The chip was then plasma cleaned for an additional 20 s to ensure the particles had adhered to the window and would not wash out during liquid flow. The holder was loaded and leak‐checked by flowing ethanol through and first visually checking for leaks and then by using a vacuum pump (Hummingbird Scientific Pfeiffer vacuum DCU) to obtain a pressure of 10^−6^ mbar. On completion, the holder was transferred to a prealigned TEM and connected to a syringe pump. Final beam alignments (e.g. probe current) were made with ethanol in the cell.

LCTEM data was collected using an FEI Titan^3^ Themis G2 S/TEM operated at 300 kV with an FEI Super‐X EDX system and a Gatan OneView CCD. A *ca*. 1.4 Å probe was formed for STEM with an estimated convergence semi‐angle of *ca*. 10 mrad (limited by the second smallest C3 aperture) and a probe current of up to 150 pA for LC STEM (as measured by the dose meter on the flu cam, calibrated using a Faraday cup). Quoted electron fluences (*F*) were calculated using Equation (1) where *t* is the dwell time, *I* is the probe current in Amps, *e* is the magnitude of the charge of an electron (1.602 × 10^−19^ C) and *d_s_* is the pixel size. Typical dwell times ranged from 5 to 20 μs and the pixel size varied with magnification (typically of the order of nm). In addition, the electron flux (averaged over the frame) *F*_av_ was calculated using Equation (2) where *A* is the frame area in Å^2^.

(1) $$F\left(e^{-}/{\dot{A}}^{2}\right)=\frac{I\times t}{e\times d_{s}^{2}},$$

(2) $$F_{av}\left(e^{-}/{\dot{A}}^{2}\cdot s\right)=\frac{I}{eA}.$$

### 5.3 Raman spectroscopy

A LabRAM HR evolution confocal Raman microscope was used with a 488 nm laser, an 1800 mm^−1^ grating and 50× objective. Reference spectra were collected from a LC containing only the reaction solution (9:1 [12 mM CaSO_4_(aq)]:[ethanol]), and bassanite and gypsum dried onto glass slides (Figure S7). The acquisition time for the in situ reaction was three accumulations of 30 s at 50% power giving a time resolution of 90 s.

### 5.4 Cryo‐TEM

Cryo‐TEM experiments were performed as follows: Using an FEI Vitrobot©, 3.5 μl of the mixed (bassanite and 9:1 [12 mM CaSO_4(aq)_]:[ethanol]) solution was loaded onto a Quantifoil grid (EM resolutions) and plunge frozen in liquid ethane. This allowed snap shots of the reaction to be captured at differing time points. Manual preparation via the Vitrobot was limited to time points of >20 s. In order to probe shorter reaction times, time‐resolved cryo‐TEM was performed. Grids for time‐resolved cryo‐EM were prepared using a home‐built instrument for plunge freezing described in a previous publication by Kontziampasis *et al*.^39^ Spray‐based sample application was done using gas dynamic virtual nozzles in spraying mode. Two different grid preparation set‐ups were used as described previously in a further publication by Klebl *et al*.^40^

#### 5.4.1 On‐grid mixing

Quantifoil 300 mesh Cu R2/2 grids were glow‐discharged in a Cressington 208 carbon coater with glow discharge unit at 0.1 mbar air, 15 mA for 99 s. Three microlitres of the bassanite in ethanol mixture (diluted fourfold in ethanol) were applied to each grid, blotted and allowed to dry on filter paper. Grids with dried bassanite crystals were held by a pair of Dumount N5 tweezers which were mounted on the device. The grid was then passed through a spray (2.6 μl/s, 2 bar N_2_ spray gas) of 9:1 [12 mM CaSO_4_(aq)]:[ethanol] at ∼ 1 m/s. Thirty milliseconds after the grid had passed, the spray was stopped. The mixture (bassanite crystals and aqueous CaSO_4_) was then incubated on‐grid for a defined time (0.9 s, 4.9 s, 9.9 s and 19.9 s) in the environmental chamber of the device at ambient temperature (20°C) and high humidity (70–90%). After this reaction time, the reaction was stopped by plunging the grid into liquid ethane. The time for sample application, acceleration, deceleration and freezing was ∼ 0.1 s, giving final time delays of 1 s, 5 s, 10 s and 20 s.

#### 5.4.2 In‐flow mixing

For in‐flow mixing, Quantifoil 400 mesh Cu R1.2/1.3 grids were used after glow discharge in a PIE Scientific Tergeo Plasma Cleaner with remote plasma, 15 W RF power, in a 1:1:1.5 mixture of nitrogen/oxygen/argon for 1 min. Fourfold diluted bassanite in ethanol and 9:1 [12 mM CaSO_4_(aq)]:[ethanol] was loaded into two separate glass syringes which were subsequently moved at a flow rate of 4.2 μl/s and the two reactants met in an Upchurch High Pressure Static Mixing Tee, with a total flow rate of 8.3 μl/s. The reaction took place in‐flow while the liquid was in the delay line, which was 25 cm in length and had a 381 μm inner diameter (28.5 μl volume). We assume laminar flow in the delay line, which leads to a distribution of timepoints. Grids were prepared at 4 timepoints with the in‐flow mixing setup (2.7 s, 5 s, 15 s and 20 s). For the 2.7 s timepoint, samples were mixed and continuously flowed through the delay line. The time between spraying and vitrification was negligible (0.02–0.03 s). The resulting median residence time was 2.7 s, with a predicted 87 % of particles between 2 and 5 s. For the 5 s, 15 s and 20 s timepoints, the flow was stopped for 2.5 s, 12.5 s and 17.5 s, to give median time‐delays of 5.2 s, 15.2 s and 20.2 s, respectively, with 89% of particles between 4.5–8 s, 14.5–18 s and 19.5–23 s, respectively.

All time‐resolved cryo grids were screened and imaged using an FEI Titan Krios G3i, X‐FEG and autoloader operating at 300 kV and equipped with an FEI Ceta and FEI Falcon E3C direct electron detectors.

## Supporting information

Supplementary Information

Supplementary video 1

Supplementary video 2

Supplementary video 3

Supplementary video 4
