# Supplementary material for: Evaluation of correlated studies using liquid cell and cryo‐transmission electron microscopy: Hydration of calcium sulphate and the phase transformation pathways of bassanite to gypsum
Source: J Microsc. 2022 Apr 18;288(3):155–68. doi: 10.1111/jmi.13102 (PMC10084335; doi:10.1111/jmi.13102)
Supplement: Supplementary file 1 — Supplementary Information [file JMI-288-155-s001.docx]

Evaluation of correlated studies using liquid cell- and cryo-transmission electron microscopy (TEM): Hydration of Calcium Sulfate and the Phase Transformation Pathways of Bassanite to Gypsum.

M. Ilett^1*^, H.M. Freeman^1^, Z. Aslam^1^, J. M. Galloway^2^, D. P. Klebl^3^, S. P. Muench^3^, I. McPherson^4^, O. Cespedes^5^, Y-Y. Kim^2^, F.C. Meldrum^2^, S.R. Yeandel^6^, C. Freeman^6^, J. Harding^6^, R.M.D. Brydson^1^

1. School of Chemical and Process Engineering, University of Leeds, Leeds, LS2 9JT, UK
2. School of Chemistry, University of Leeds, Leeds, LS2 9JT, UK
3. School of Biomedical Sciences and Astbury Centre for Structural and Molecular Biology, University of Leeds, Leeds, LS2 9JT, UK
4. Department of Chemistry, University of Warwick, Gibbet Hill, Coventry, CV4 7AL
5. Department of Physics, University of Leeds, Leeds, LS2 9JT, UK
6. Department of Materials Science and Engineering, University of Sheffield, Sheffield S1 3JD.

*corresponding author: m.a.ilett@leeds.ac.uk

**SUPPLEMENTARY INFORMATION**

**RESULTS**


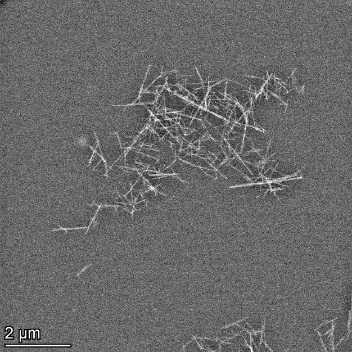


0 s


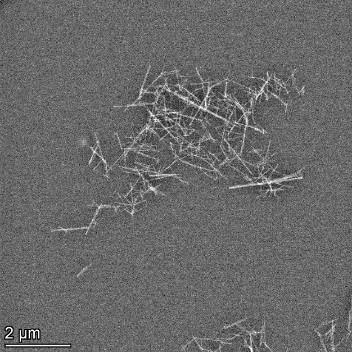


250 s

**Figure S1**: Bassanite nanorod precursor was stable under the electron beam when dispersed in ethanol. Total accumulated electron fluence after 250 s was 23 e^-^/Å^2^.


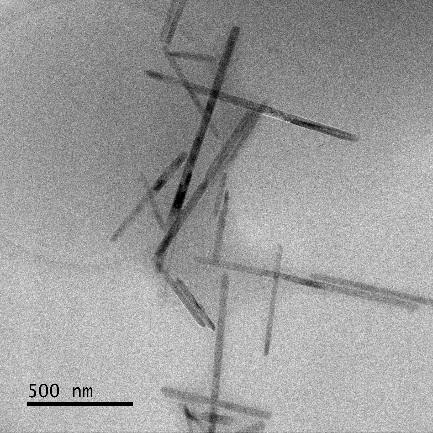

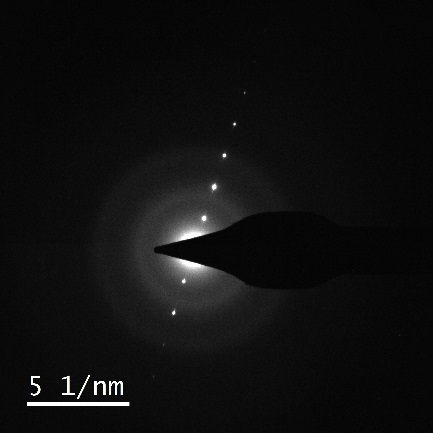

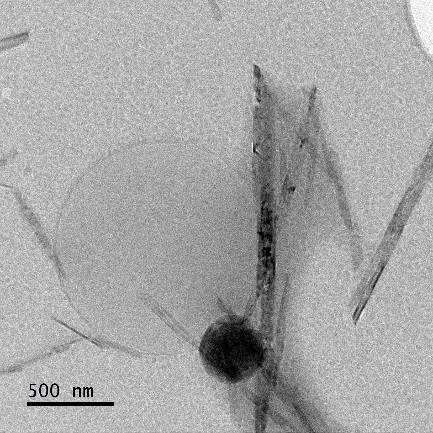


6 Å


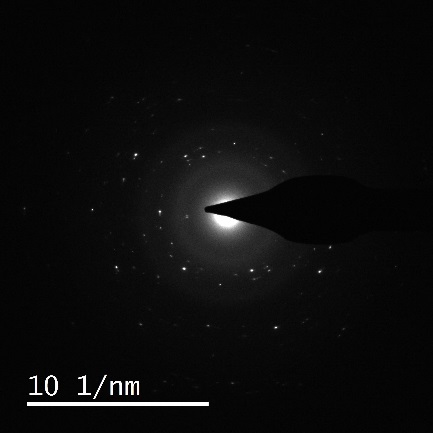


(A)

(B)

**Figure S2**: (A) Cryo-TEM images and SAED of: (A) Bassanite precursor in ethanol, distinctive ~6 Å d-spacing observed in the SAED pattern taken from particle shown by the white arrow and (B) image taken after mixing the bassanite precursor with the aqueous CaSO_4_ solution for 20 s. Evidence that the particles had started to transform to gypsum with the SAED pattern showing distinctive rings at ~4.2 Å (dashed) and ~3 Å (solid).

(A)

(B)

(C)

**Figure S3**: (A) Directionality plot averaged from cryo-TEM images taken after 5, 10 and 20 s for on-grid mixing. (B) Directionality plot averaged from cryo-TEM images taken after 2.7, 5, 15 and 20 s for in-flow mixing. (C) Directionality plot comparing directionality after 5 s for in-flow and on-grid mixing. The plot shows little evidence of alignment within the images for either set up. The error is plotted as the standard deviation of the directionality in n >10 images.


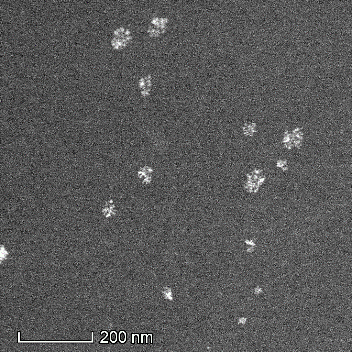

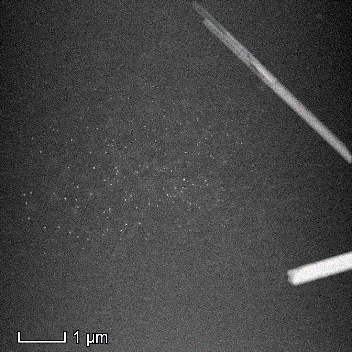


**Figure S4**: STEM image of the electron beam induced formation of amorphous CaSO_4_ after flowing through a 9:1 [15 mM CaSO_4_(aq)]:[ethanol] solution. These amorphous ‘blobs’ formed quicker under the beam when using a 15 mM CaSO_4_ aqueous solution compared to 12 mM.

Bassanite {0 0 1} plane


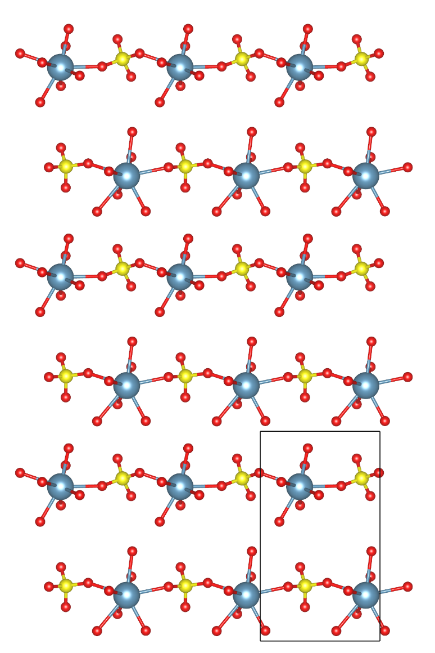

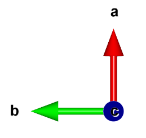


7.00 Å

7.43 Å

7.07 Å

7.07 Å

6.69 Å

7.00 Å


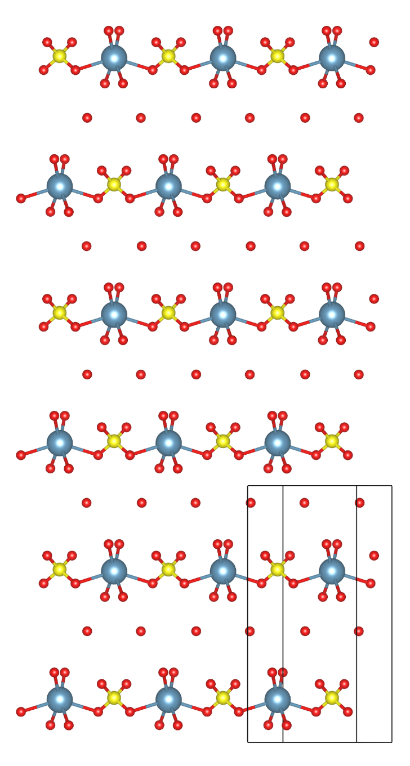

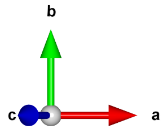


8.35 Å

6.52 Å

8.35 Å

8.34 Å

6.52 Å

8.35 Å

Gypsum {0 0 1} plane

**Figure S5**: Molecular dynamic simulation of bassanite and gypsum from average structures taken at 300 K. Both show a hexagonal motif between the calcium and sulfate ions, and chains of Ca-SO_4_ perpendicular to {001} suggesting some degree of epitaxy between the (001) planes in bassanite and gypsum. Gypsum is elongated along the b axis by ~25% and shortened along the a axis by ~ 7%.

**MATERIALS AND METHODS**


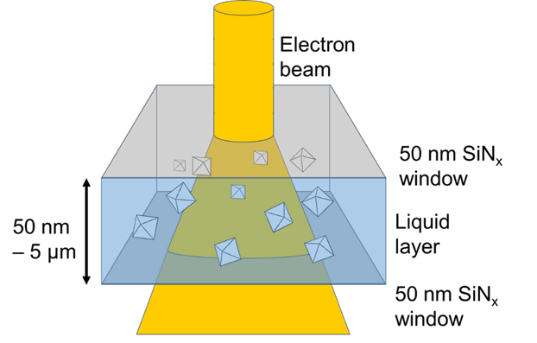


**Figure S6**: Schematic of a typical liquid cell TEM set-up

**Figure S7**: Reference Raman spectra for a liquid cell containing only the reaction solution (9:1 [12 mM CaSO_4_(aq)]:[ethanol]) showing no peaks, and reference spectra of bassanite and gypsum on glass slides showing the distinctive shift of the υ_1_SO_4_^2-^ peak from 1016 cm^-1^ to 1008 cm^-1^ respectively.
